# Ghrelin ameliorates tumor-induced adipose tissue atrophy and inflammation *via* Ghrelin receptor-dependent and -independent pathways

**DOI:** 10.18632/oncotarget.27705

**Published:** 2020-09-01

**Authors:** Haiming Liu, Jiaohua Luo, Bobby Guillory, Ji-an Chen, Pu Zang, Jordan K. Yoeli, Yamileth Hernandez, Ian (In-gi) Lee, Barbara Anderson, Mackenzie Storie, Alison Tewnion, Jose M. Garcia

**Affiliations:** ^1^Geriatric Research, Education and Clinical Center, Veterans Affairs Puget Sound Health Care System, Seattle, WA, USA; ^2^Gerontology and Geriatric Medicine, University of Washington Department of Medicine, Seattle, WA, USA; ^3^Division of Endocrinology, Diabetes and Metabolism, MCL, Center for Translational Research on Inflammatory Diseases, Michael E. DeBakey Veterans Affairs Medical Center, Department of Medicine, Baylor College of Medicine, Houston, TX, USA; ^4^Department of Environmental Hygiene, College of Preventive Medicine, Army Medical University, Chongqing, China; ^5^Department of Health Education, College of Preventive Medicine, Army Medical University, Chongqing, China; ^6^Department of Endocrinology, Nanjing Jinling Hospital, Nanjing, China; ^*^These authors contributed equally to this work

**Keywords:** cachexia, cancer, muscle, ghrelin, adipose tissue

## Abstract

Adipose tissue (AT) atrophy is a hallmark of cancer cachexia contributing to increased morbidity/mortality. Ghrelin has been proposed as a treatment for cancer cachexia partly by preventing AT atrophy. However, the mechanisms mediating ghrelin’s effects are incompletely understood, including the extent to which its only known receptor, GHSR-1a, is required for these effects. This study characterizes the pathways involved in AT atrophy in the Lewis Lung Carcinoma (LLC)-induced cachexia model and those mediating the effects of ghrelin in *Ghsr*^+/+^ and *Ghsr*^–/–^ mice. We show that LLC causes AT atrophy by inducing anorexia, and increasing lipolysis, AT inflammation, thermogenesis and energy expenditure. These changes were greater in *Ghsr*^–/–^. Ghrelin administration prevented LLC-induced anorexia only in *Ghsr*^+/+^, but prevented WAT lipolysis, inflammation and atrophy in both genotypes, although its effects were greater in *Ghsr*^+/+^. LLC-induced increases in BAT inflammation, WAT and BAT thermogenesis, and energy expenditure were not affected by ghrelin. In conclusion, ghrelin ameliorates WAT inflammation, fat atrophy and anorexia in LLC-induced cachexia. GHSR-1a is required for ghrelin’s orexigenic effect but not for its anti-inflammatory or fat-sparing effects.

## INTRODUCTION

Cachexia, defined as an involuntary loss of muscle and adipose tissue, is present in 15–65% of cancer patients depending on the tumor type, and it is strongly associated with poor outcomes [1, 2]. Adipose tissue, once considered only a high-energy fuel reserve, has emerged recently as an active metabolic organ modulating inflammation, energy expenditure and food intake in non-cancer conditions [3]. Accelerated loss of adipose tissue plays an important role in cancer cachexia contributing significantly to the increased morbidity and mortality [4].

Increased inflammation is common in cancer [5] and is associated with adipose tissue wasting in human studies [6]. White adipose tissue (WAT) is a significant source of inflammatory cytokines accounting for more than 30% of circulating interleukin (IL)-6 [7] and this and other inflammatory cytokines have been linked to WAT atrophy in cancer [8–10]. Also, a phenotypic switch from WAT to brown adipose tissue (BAT) known as “browning” is thought to contribute to the overall increase in energy expenditure and WAT atrophy seen in cancer cachexia [10]. Nevertheless, the mechanisms regulating adipose tissue atrophy and dysfunction in cancer cachexia are incompletely understood.

Ghrelin, originally identified as the endogenous ligand for the growth hormone secretagogue receptor (GHSR)-1a, has emerged as a pleiotropic hormone that regulates body weight, body composition and energy expenditure [11]. In non-cancer models, it has been shown to increase food intake by activating neuropeptide Y and agouti-related peptide-secreting neurons in the hypothalamus and to have direct effects on adipocytes [11–13]. Ghrelin has also been proposed as a promising target for cancer cachexia and it has been shown to prevent fat atrophy in tumor-bearing animals and in patients with cancer cachexia [14–16]. However, the mechanisms mediating these effects are incompletely understood. Interestingly, emerging data suggest that some of these effects are independent of the only ghrelin receptor identified to date, GHSR-1a [17, 18]. Ghrelin prevents C2C12 myotube atrophy induced by cisplatin in the absence of GHSR-1a [16]. In addition, an in-vivo study showed that ghrelin prevents fasting-induced muscle atrophy and improve protein synthesis in Ghsr-1a knockout (KO, *Ghsr*^−/−^) mice [19]. To our knowledge, the extent to which ghrelin’s effects on adipose tissue atrophy and metabolism in cancer cachexia are mediated by GHSR-1a is not known. Many studies have reported that *Ghsr*^−/−^ mice have normal growth rates and normal appetite under conditions of standard laboratory housing [20–22]. However, under prolonged fasting, *Ghsr*^−/−^ showed lower glucose and insulin levels [23]. With aging (18-22 months old), *Ghsr*^−/−^ mice showed higher levels of energy expenditure and resting metabolic rate but food intake and locomotor activity were similar to that of old wild type (WT) mice [24].

Lewis Lung Carcinoma (LLC) is a murine tumor cell line that induces cachexia and decreases food intake in C57BL/6J mice [16, 25, 26] with a relatively high tumor burden (3–5 g) [16, 25]. This animal model has been widely used for cancer cachexia studies [25] as it exhibits several key features of cachexia including anorexia, weight loss, loss of lean and fat mass and increased energy expenditure [16, 27–30]. Since our GHSR-1a WT (*Ghsr*^+/+^) and KO (*Ghsr*^−/−^) animals have been developed in a C57BL/6J background, this model is particularly well-suited to determine the role of ghrelin and GHSR-1a in tumor-induced cachexia. The objectives of the current study were: 1) to characterize the pathways involved in adipose tissue atrophy in the LLC-induced cachexia model; and 2) to determine the pathways mediating the effects of ghrelin on adipose tissue and the relative contribution of GHSR-1a. We tested the effect of LLC tumor cell implantation with and without exogenous ghrelin administration in GHSR-1a WT (*Ghsr*^+/+^) and KO (*Ghsr*^−/−^) mice using heat-killed (HK)-LLC cells-inoculated mice of both genotypes as negative controls.

## RESULTS

We utilized C57BL/6J congenic mice with (*Ghsr*^+/+^) or without GHSR-1a (*Ghsr*^−/−^). Five- to seven-month-old male *Ghsr*^+/+^ and *Ghsr*^−/−^ mice were inoculated with 1 × 10^6^ heat-killed (HK, control) or live LLC cells in the right flank. When the tumor was palpable (approximately 1 wk after implantation), tumor-bearing mice were injected with vehicle (saline solution, tumor-vehicle, T+V) or ghrelin (0.8 mg/kg, tumor-ghrelin, T+G) subcutaneously (s.q.) twice/day, while heat-killed mice were injected with vehicle (HK+V) until the end of the experiments (2 weeks after the tumor became palpable). Body weight and fat mass were measured by nuclear magnetic resonance (NMR) before tumor implantation (baseline), and 2 weeks after tumors were noted. Brown adipose tissue (BAT) and inguinal and epididymal white adipose tissue (iWAT, eWAT) were collected and weighed upon sacrificing animals 2 weeks after tumors were noted. We confirmed that *Ghsr*^−/−^ mice did not express *Ghsr* globally by genotyping. Also, there was no expression of *Ghsr* in iWAT or BAT on either genotype (Supplementary Figure 1).

### Ghrelin prevents tumor-induced weight loss and adipose tissue atrophy only partially *via* GHSR-1a

**Figure 1:**
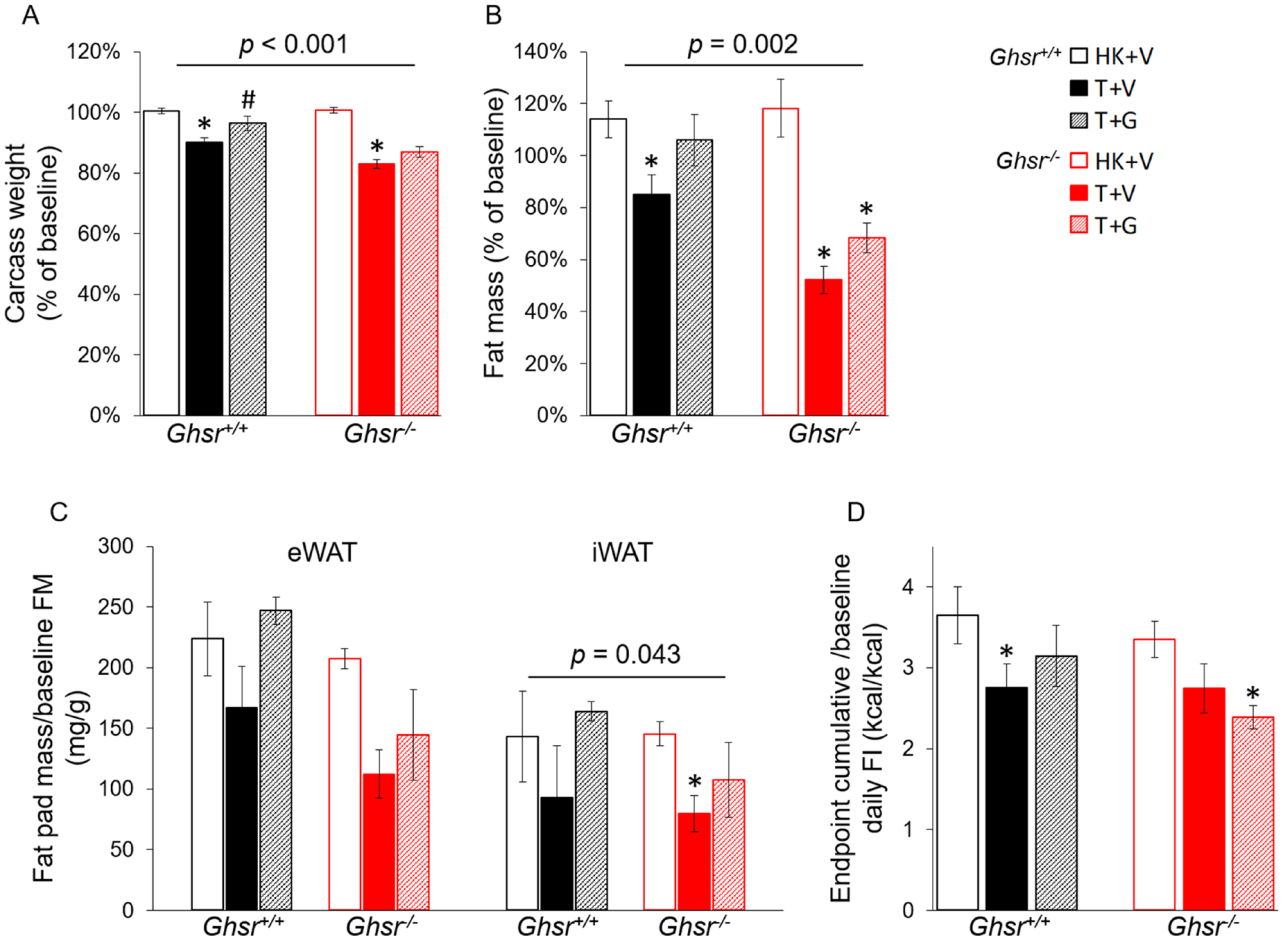
Effects of ghrelin on body weight, fat mass, and food intake in LLC-induced cachexia. HK+V: heat-killed + vehicle; T+V: tumor + vehicle; T+G: tumor + ghrelin. Changes in (**A**) body weight (carcass weight, body weight at endpoint without tumor, *n* = 8–10) expressed as % change from baseline body weight (before tumor implantation) and (**B**) fat body mass by NMR expressed as % change from baseline (fat mass by NMR before tumor implantation, *n* = 8–10). (**C**) Fat pad mass normalized to baseline NMR fat mass (mg/g, *n* = 4–6). (**D**) Average cumulative food intake (FI) normalized to baseline daily FI [kcal/kcal, 72 h cumulative FI during CLAMS before sacrificing/baseline daily FI (before LLC implantation), *n* = 4–6). Two-way ANOVA was performed to detect genotype and treatment differences. ^*^ *p* < 0.05 compared to HK+V within the same genotype. ^#^ *p* < 0.05 compared to T+V within the same genotype. Genotype effects are shown in *p*-values above the corresponding figures (*p* < 0.05). Data are shown as mean ± SE.

LLC tumor implantation induced significant decreases in carcass weight in both genotypes; although, the decrease was more profound in *Ghsr*^−/−^ than in *Ghsr*^+/+^ mice (Figure 1A, genotype effect: *p* < 0.001). The same pattern was seen in whole body fat mass measured by NMR (Figure 1B, genotype effect: *p* = 0.002) as well as in iWAT and eWAT pad weights measured upon dissection (Figure 1C, genotype effect on iWAT: *p* = 0.043). These changes were attenuated by ghrelin administration in *Ghsr*^+/+^ and *Ghsr*^−/−^ tumor-bearing animals. Tumor implantation induced a decrease in food intake (FI) in *Ghsr*^+/+^ mice that was partially attenuated by ghrelin treatment (Figure 1D). In *Ghsr*^−/−^ mice, FI was lower in tumor bearing mice though differences only reached significance for T+G mice (Figure 1D). There was no difference in tumor mass between groups (*Ghsr*^+/+^: T+V: 3.7 ± 0.5 g; T+G: 3.5 ± 0.5 g; *Ghsr*^−/−^: T+V: 4.4 ± 0.6 g; T+G: 3.0 ± 0.3 g. Two-way ANOVA: Genotype effect: *p* = 0.872; Treatment effect: *p* = 0.115).

### Ghrelin prevents adipose tissue lipolysis by modulating ATGL and HSL *via* GHSR-1a-dependent and independent pathways, respectively

**Figure 2:**
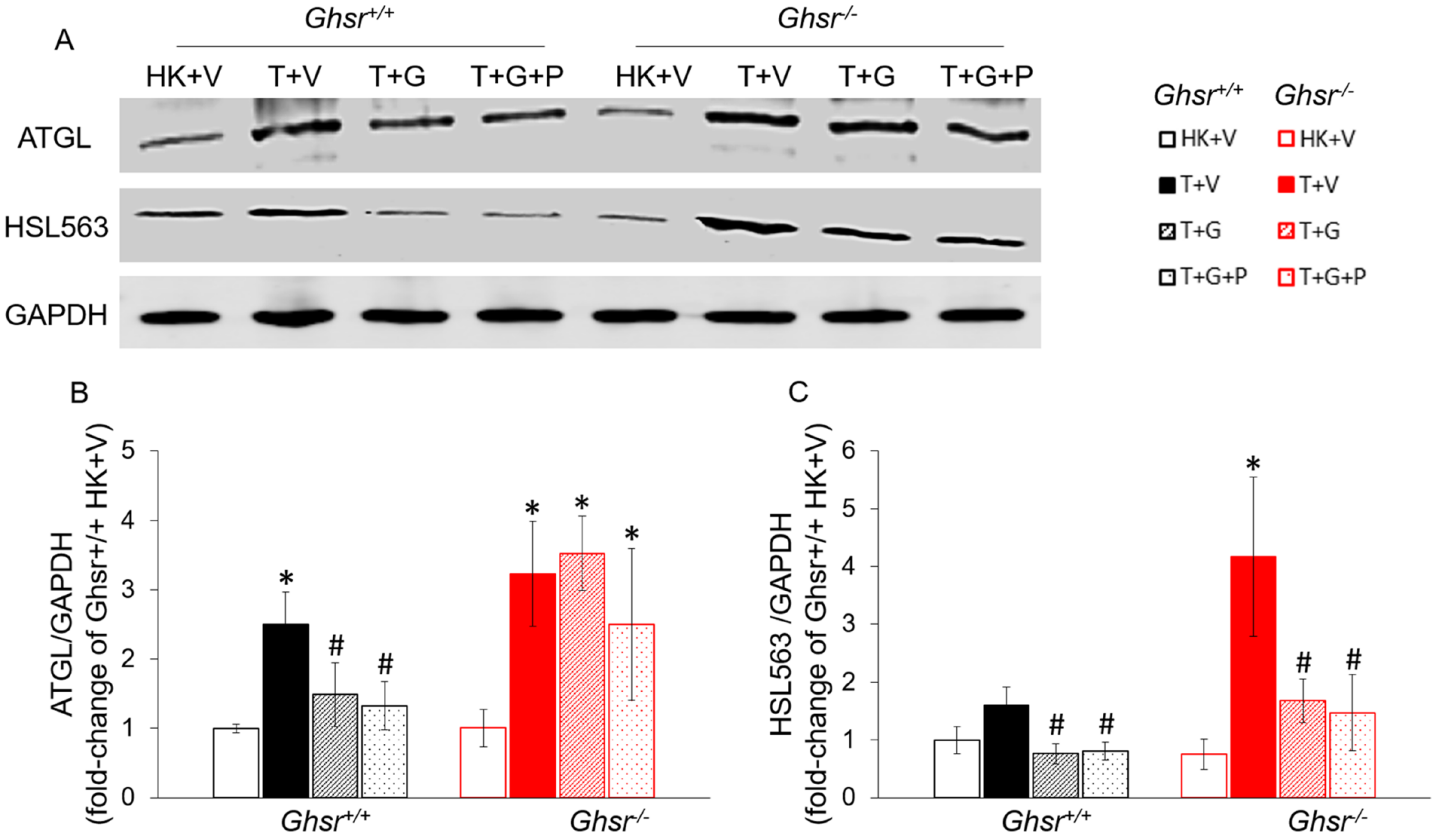
Effects of ghrelin on LLC-induced protein-level changes in lipases in iWAT. HK+V: heat-killed + vehicle; T+V: tumor + vehicle; T+G: tumor + ghrelin; T+G+P: tumor + ghrelin + pair-fed (**A**) Representative Western blots for ATGL, HSL, and GAPDH. (**B**) ATGL expression in each group. (**C**) HSL expression in each group. Western blots were quantified by densitometry and normalized to GAPDH. Results are presented as fold change of *Ghsr*^+/+^ HK+V. Two-way ANOVA was performed to detect genotype and treatment differences. ^*^ *p* < 0.05 compared to HK+V within the same genotype. ^#^ *p* < 0.05 compared to T+V within the same genotype. No genotype difference was detected. Data are shown as mean ± SE. *n* = 4/group.

To assess lipolysis, we measured levels of phosphorylated Ser563 hormone sensitive lipase (HSL) and adipose triglyceride lipase (ATGL) protein levels. For this experiment, we also included groups of *Ghsr*^+/+^ and *Ghsr*^−/−^ tumor-bearing animals treated with ghrelin that were pair-fed (T+G+P) to tumor-bearing animals receiving vehicle (T+V) to exclude a possible effect of anorexia. As shown in Figure 2A and 2C, tumor implantation increased ser563 pHSL levels and this increase was more profound in *Ghsr*^−/−^ than in *Ghsr*^+/+^ mice. In the presence of GHSR-1a, these changes were prevented by ghrelin independently of food intake. Ghrelin partially prevented this increase in the absence of GHSR-1a (Figure 2C). ATGL protein levels were also increased by LLC implantation. Interestingly, ghrelin prevented these changes independently of food intake but in a GHSR-1a-dependent manner (Figure 2B).

### Ghrelin attenuates tumor-induced inflammation in iWAT but not in iBAT or in circulation

**Figure 3:**
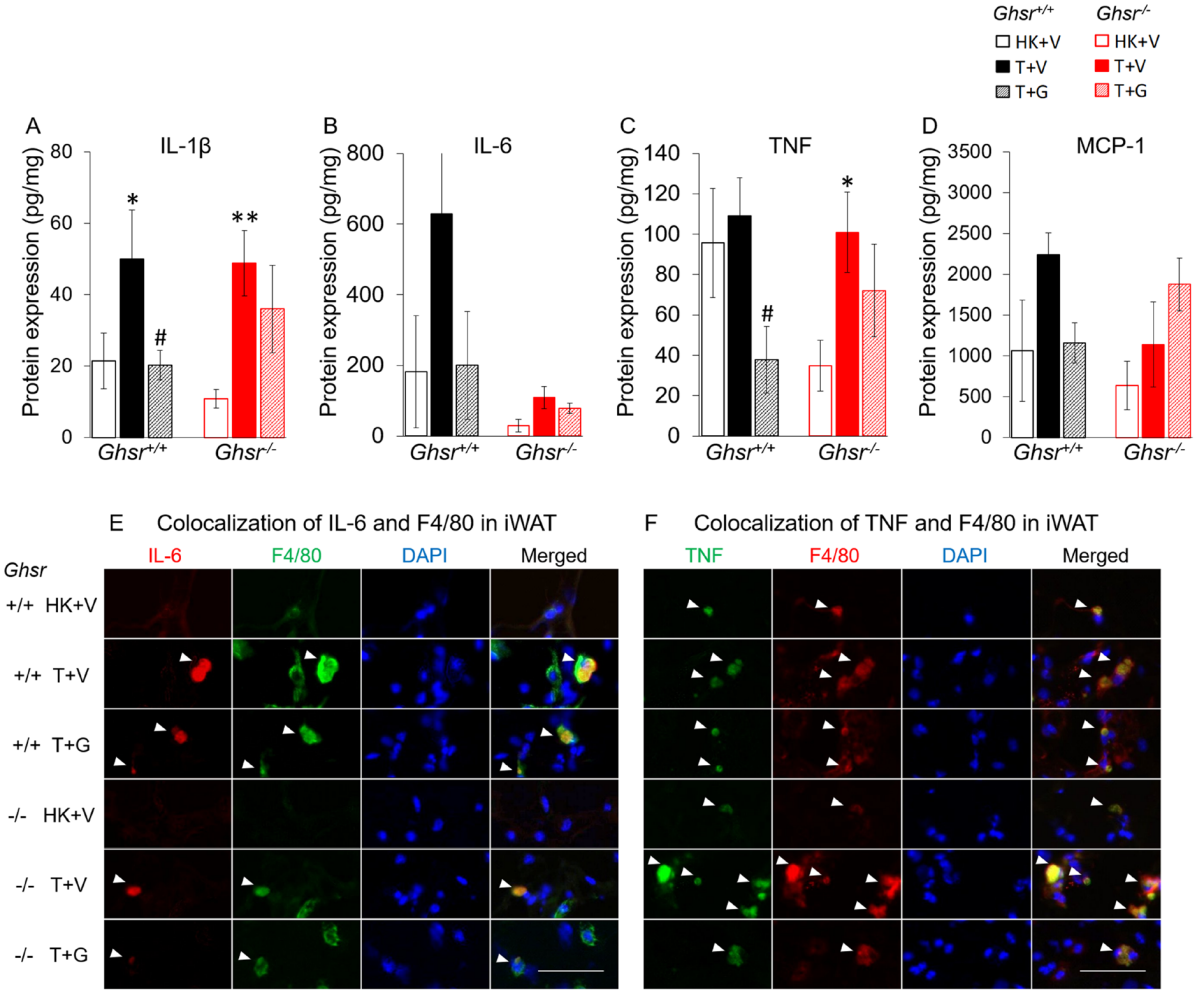
Effects of ghrelin on LLC-induced changes in inflammation and macrophages in iWAT. HK+V: heat-killed + vehicle; T+V: tumor + vehicle; T+G: tumor + ghrelin. Protein levels of inflammatory markers (**A**) IL-1β, (**B**) IL-6, and (**C**) TNF; and (**D**) macrophage marker MCP-1 in iWAT (pg/mg). Two-way ANOVA was performed to detect genotype and treatment differences. ^*^ *p* < 0.05; ^**^ *p* < 0.01 compared to HK+V within the same genotype. ^#^ *p* < 0.05 compared to T+V within the same genotype. No genotype difference was detected. Data are shown as mean ± SE. *n* = 6–7/group. (**E** and **F**) Colocalization of inflammation and macrophages in iWAT. (E) Representative images of colocalization of inflammatory marker IL-6 and macrophage marker F4/80 in iWAT (IL-6 in Texas red; F4/80 in FITC green; nuclei in DAPI blue). (F) Representative images of colocalization of inflammatory marker TNF and macrophage marker F4/80 in iWAT (TNF in FITC green; F4/80 in Texas red; nuclei in DAPI blue). Positively stained inflammatory markers and colocalizations with macrophages are indicated by white arrows. Scale bars, 50 μm.

In *Ghsr*^+/+^ animals, protein level for the pro-inflammatory cytokines Interleukin (IL)-1β in iWAT were increased in tumor-bearing mice and ghrelin prevented this increase (Figure 3A). Ghrelin also decreased tumor necrosis factor (TNF) level in *Ghsr*^+/+^ tumor bearing mice although there was no significant difference between heat-killed+vehicle (HK+V) *vs*. tumor+vehicle (T+V) groups (Figure 3C). IL-6 and the macrophage marker monocyte chemoattractant protein-1 (MCP-1), a key chemokine responsible for migration and infiltration of monocytes/macrophages [31], followed a similar pattern although the differences did not reach statistical significance (Figure 3B and 3D). Interestingly, in *Ghsr*^−/−^ mice, LLC-induced IL-6 level increases in iWAT appear to be dampened; whereas, MCP-1 levels were not affected by LLC or by ghrelin. Immunohistochemistry staining showed complete co-localization of IL-6 and TNF with F4/80, a marker of macrophages in mice, demonstrating that the source of these cytokines in iWAT is macrophages (Figure 3E and 3F).

**Figure 4:**
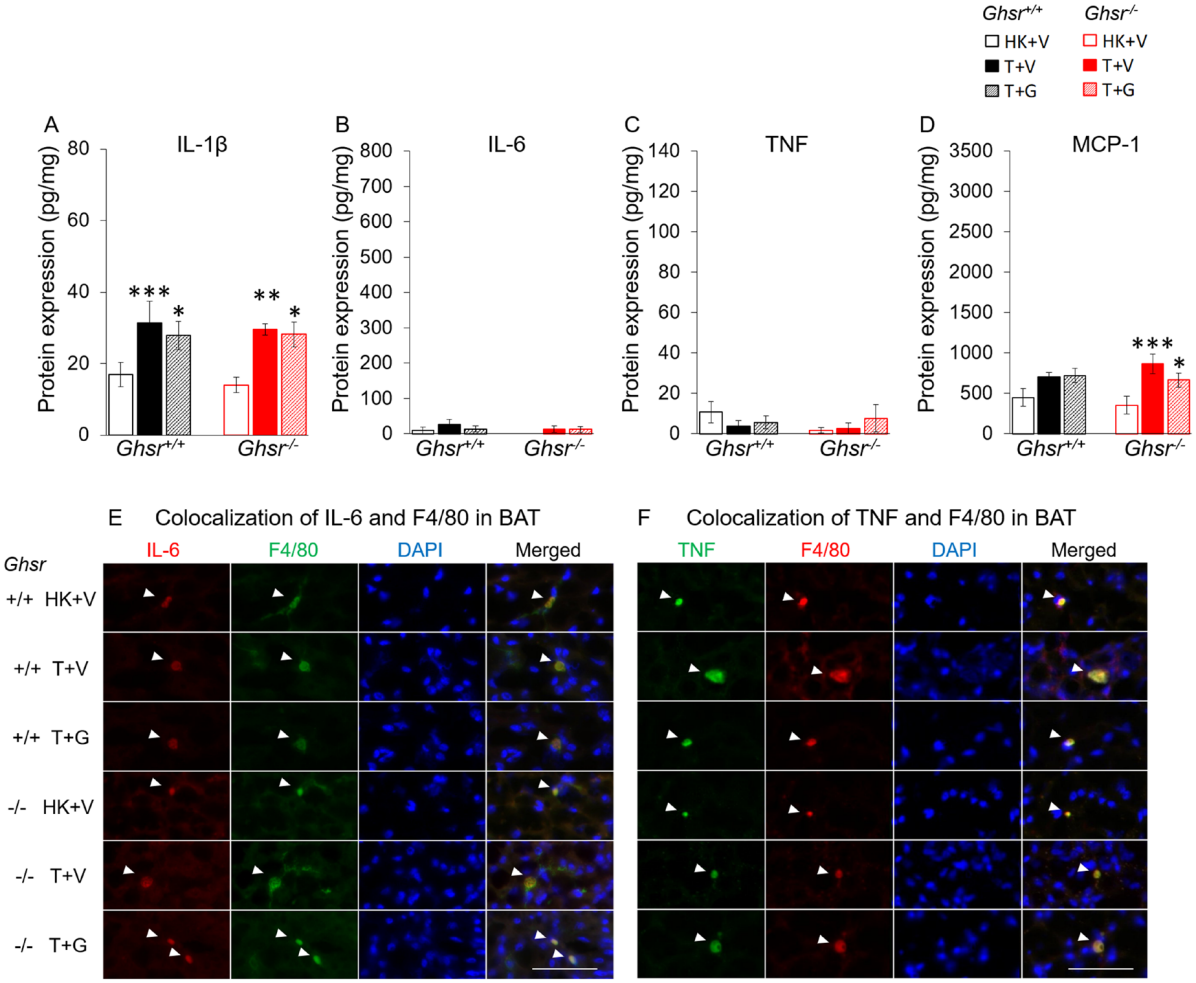
Effects of ghrelin on LLC-induced changes in inflammation and macrophages in BAT. HK+V: heat-killed + vehicle; T+V: tumor + vehicle; T+G: tumor + ghrelin. Protein levels of inflammatory markers (**A**) IL-1β, (**B**) IL-6, and (**C**) TNF; and (**D**) macrophage marker MCP-1 in BAT (pg/mg). Two-way ANOVA was performed to detect genotype and treatment differences. ^*^ *p* < 0.05; ^**^ *p* < 0.01; ^***^ *p* < 0.001 compared to HK+V within the same genotype. ^#^ *p* < 0.05; ^###^ *p* < 0.001 compared to T+V within the same genotype. No genotype difference was detected. Data are shown as mean ± SE. *n* = 6–7/group. (**E** and **F**) Colocalization of inflammation and macrophages in BAT. (E) Representative images of colocalization of inflammatory marker IL-6 and macrophage marker F4/80 in BAT (IL-6 in Texas red; F4/80 in FITC green; nuclei in DAPI blue). (F) Representative images of colocalization of inflammatory marker TNF and macrophage marker F4/80 in BAT (TNF in FITC green; F4/80 in Texas red; nuclei in DAPI blue). Positively stained inflammatory markers and colocalizations with macrophages are indicated by white arrows. Scale bars, 50 μm.

In BAT, all the inflammatory markers were generally lower than in WAT. IL-1β was increased in both genotypes (Figure 4A) and MCP-1 only in *Ghsr*^−/−^ (Figure 4D). Ghrelin did not significantly affect these changes. IL-6 and TNF levels were not significantly different between groups (Figure 4B–4C). Nevertheless, immunohistochemistry analysis shows similar results as in iWAT suggesting that IL-6 and TNF in BAT were also derived exclusively from macrophages (Figure 4E–4F). Plasma cytokine and MCP-1 levels followed a different pattern than those seen in adipose tissue being increased by LLC and not modified by ghrelin (Supplementary Figure 2).

### Ghrelin does not prevent the increases in UCP-1 induced by LLC in iWAT or BAT

**Figure 5:**
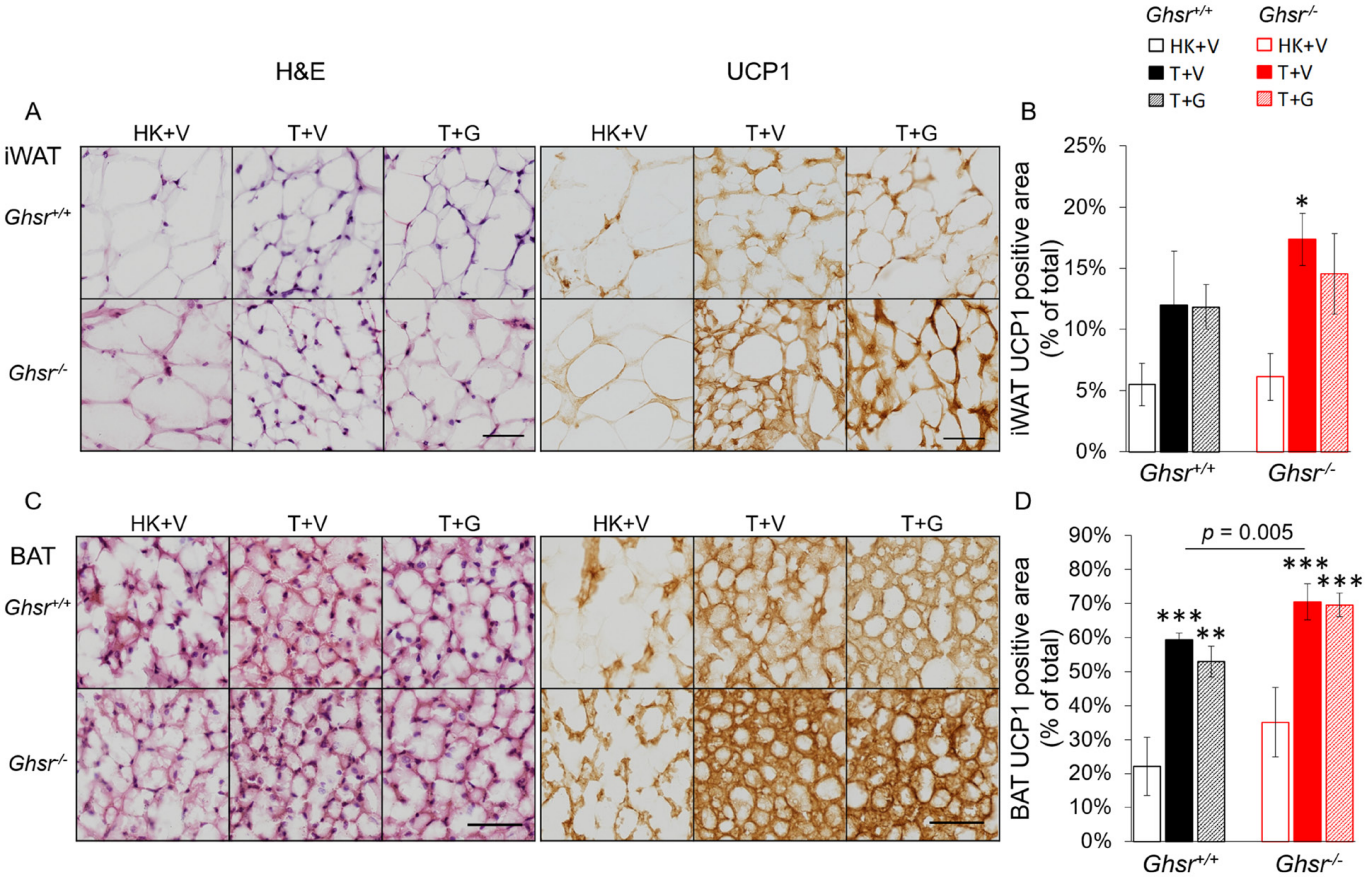
Expression of UCP-1 in iWAT and BAT. HK+V: heat-killed + vehicle; T+V: tumor + vehicle; T+G: tumor + ghrelin. (**A**) Representative H&E and IHC images of UCP-1 in iWAT. (**B**) UCP-1 positive area is expressed as % of the total analyzed area in iWAT (*n* = 4–6). (**C**) Representative H&E and IHC images of UCP-1 in BAT. (**D**) UCP-1 positive area is expressed as % of the total analyzed area in BAT (*n* = 4–6). Two-way ANOVA was performed to detect genotype and treatment differences. ^*^ *p* < 0.05; ^**^ *p* < 0.01; ^***^ *p* < 0.001 compared to HK+V within the same genotype. Genotype effects are shown as *p*-values above the corresponding figures (*p* < .05). Data are shown as mean ± SE. Scale bars, 50 μm.

Thermogenesis in BAT is activated *via* uncoupling protein-1 (UCP-1) by de-coupling oxidative phosphorylation from ATP synthesis and dissipating heat in the inner mitochondrial membrane [32]. A similar process has been reported in WAT which has been described as “fat browning” with transformation of “white” to “beige” adipocytes [33, 34]. To test the effect of LLC and the role of ghrelin and GHSR-1a on this pathway, we quantified UCP-1 levels in iWAT and BAT using immunohistochemistry (IHC) by normalizing the positively-stained area to the total cross-sectional area of the adipose tissue. Tumor implantation induced increases in UCP-1 expression in iWAT and BAT in both genotypes and these increases were more pronounced in *Ghsr*^−/−^ than in *Ghsr*^+/+^ (Figure 5A–5D). In iWAT, the LLC-induced UCP-1 increase only reached significance in the tumor-bearing *Ghsr*^−/−^ mice and no significant effect of ghrelin was observed (Figure 5A–5B). In BAT, the positively stained UCP-1 area increased with tumor implantation from 22% to 59% in *Ghsr*^+/+^ and from 35% to 70% in *Ghsr*^−/−^ mice (Figure 5C–5D, genotype effect: *p* = 0.005). However, no effect of ghrelin on reducing UCP-1 in BAT was observed.

### Tumor-induced increases in energy expenditure (EE) are not prevented by ghrelin

**Figure 6:**
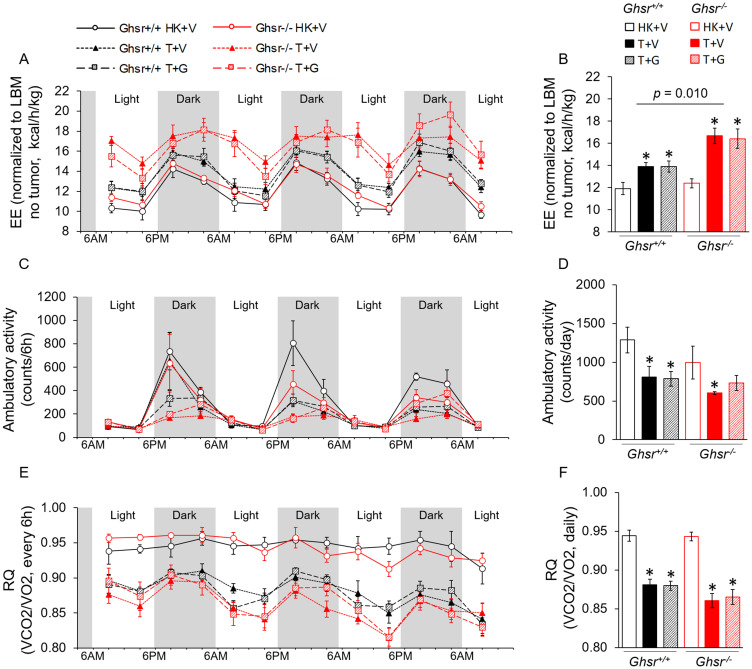
Indirect calorimetry measurements by CLAMS. HK+V: heat-killed + vehicle; T+V: tumor + vehicle; T+G: tumor + ghrelin. (**A**–**B**) Energy expenditure (EE) adjusted by LBM (without tumor) is expressed as kcal/h/kg (A) during 72 hours before sacrificing (2 weeks of ghrelin treatment); (B) average daily EE over the 72 hours before sacrificing. (**C**–**D**) Ambulatory activity (C) during 72 hours before sacrificing (counts/6 h). (D) Average daily ambulatory activity over the 72 hours before sacrificing (counts/day). (**E**–**F**) Respiratory Quotient (RQ) is calculated by VCO2/VO2 (E) during 72 hours before sacrificing (RQ/6 h); (F) average daily RQ over the 72 hours before sacrificing (RQ/day). Two-way ANOVA was performed to detect genotype and treatment differences. ^*^ *p* < 0.05 compared to HK+V within the same genotype. Genotype effects are shown in *p*-values above the corresponding figures (*p* < 0.05). *N* = 4 for HK+V groups and *N* = 6 for the rest of the groups. Data are shown as mean ± SE.

Considering that lean body mass (LBM) is the main source of heat production and tumors are measured as LBM by the NMR, we analyzed EE by normalizing to LBM without (Figure 6A–6B) and with the tumor (Supplementary Figure 3A–3B). When EE was adjusted for LBM without tumor mass (Figure 6A–6B), tumor-bearing mice showed increased EE and this difference was of greater magnitude in *Ghsr*^−/−^ animals (Figure 6A–6B; average daily EE at endpoint, genotype effect: *p* = 0.010). Animals co-administered ghrelin were not statistically different from vehicle-treated, tumor-bearing animals (Figure 6A–6B). When EE was adjusted for LBM by taking tumor mass into account (Supplementary Figure 3A–3B), no change in EE was detected in *Ghsr*^+/+^ tumor-bearing animals with or without ghrelin treatment; whereas *Ghsr*^−/−^ animals exhibited higher EE levels with tumor implantation in comparison to *Ghsr*^+/+^ (genotype effect: *p* = 0.001); although, only ghrelin-treated mice reached significance in pair-wise comparisons. This data suggests that the increases in EE seen in *Ghsr*^+/+^ animals can be explained by the tumor mass whereas this only partially explains the increases in EE seen in *Ghsr*^−/−^ mice. When energy balance was calculated by taking energy intake and expenditure into account during CLAMS, tumor implantation decreased this balance significantly more in *Ghsr*^−/−^ than in *Ghsr*^+/+^ and these changes were not significantly affected by ghrelin administration (Supplementary Figure 3C). Tumor implantation also decreased spontaneous locomotor activity (Figure 6C and 6D) and respiratory quotient (RQ) in both genotypes and ghrelin administration did not prevent these changes (Figure 6E and 6F).

## DISCUSSION

Adipose tissue atrophy is a central component of the cancer anorexia and cachexia syndrome (CACS) leading to increased morbidity and mortality [26]. Recently, emerging roles for inflammation, WAT browning and increased BAT thermogenesis have been demonstrated in CACS [10, 30, 35–41]; however, the pathways involved and their potential as therapeutic targets are not well-known. Ghrelin and agonists of its only known receptor, GHSR-1a, show potential to ameliorate CACS at least in part by preventing fat atrophy, but the specific mechanisms mediating these effects have not been fully-characterized. Given that there are no Food and Drug Administration (FDA)-approved treatments for cancer cachexia and that several clinical trials targeting this pathway have failed to meet their primary endpoints [15, 42], there is a pressing need to improve our understanding of the mechanisms of action of ghrelin in CACS. In this study, we show that ghrelin prevents LLC tumor-induced weight loss, fat atrophy and WAT inflammation and lipolysis without affecting tumor-induced BAT inflammation, WAT browning, and increased BAT uncoupling and whole-body energy expenditure. We also show that novel GHSR-1a-independent mechanisms are involved given the partial improvements in fat atrophy and WAT inflammation and lipolysis seen in ghrelin-treated, *Ghsr*^−/−^ animals. Also, this is the first report of macrophages as the source of IL-6 and TNF in both WAT and BAT in CACS.

Previously, we have shown that activation of GHSR-1a by ghrelin or GHSR-1a agonists (GHS) increases food intake and body weight [43–45]. Although here we detected similar trends for food intake, differences between groups did not reach statistical significance. We hypothesize that our study may have been underpowered to detect these differences given the greater variability of the food intake data compared to other outcomes measured. Our group and others also have shown that ghrelin reduces fat oxidation and lipolysis and increases lipogenesis and adiposity in a rodent model of cisplatin-induced cachexia by a combination of food intake-dependent and independent mechanisms [14, 16, 19]. Adipocyte lipolysis is regulated by two rate-limiting enzymes: adipose triglyceride lipase (ATGL) and hormone sensitive lipase (HSL). ATGL initiates breakdown of triacylglycerol (TAG) into diacylglycerol and HSL breaks down diacylglycerol into monoacylglycerol after being activated through phosphorylation at Ser563 by either protein kinase A (PKA) or the mitogen-activated protein kinase (MAPKs) p42 and p44 [46–48]. The critical relevance of adipose tissue lipolysis in CACS is highlighted by the fact that HSL is enhanced in adipocytes from cachectic individuals [49], and that inhibition of lipolysis through genetic ablation of ATGL or HSL protects mice from tumor-induced fat and muscle atrophy [26]. Moreover, increased lipolysis often occurs before and faster than muscle loss in CACS [50]. In the current study, we show that lipolysis, as measured by p-HSL and ATGL protein levels, was increased by tumor implantation. This was more prominent in the absence of GHSR-1a, suggesting a protective role of this endogenous pathway in tumor-bearing animals. Also, GHSR-1a mediated ghrelin’s effects on ATGL but not on HSL and this may partly explain the greater effect of ghrelin administration on preserving fat mass in *Ghsr*^+/+^ mice.

Weight loss and survival rates are correlated with IL-6 levels in cancer patients [5, 51, 52]. These observations and several mechanistic studies support the premise that inflammation plays a central role in CACS. Increases in IL-1β and TNF contribute to anorexia [2, 53, 54], and TNF and IL-6 promote lipolysis and inhibit lipogenesis in WAT leading to weight loss [38, 55–58]. Under non-cancer conditions, one third of the circulating IL-6 is produced by WAT [59] and most of this WAT-derived IL-6 comes from the stroma-vascular fraction composed of endothelial cells, monocytes/macrophages, myocytes, and fibroblasts [60], although it can also be derived from adipocytes [61]. Macrophages in WAT are known to be the source of proinflammatory cytokines in conditions leading to AT hypertrophy including obesity [62–64] but this has not been previously shown in CACS. Here we show that LLC tumor implantation induces an increase in inflammatory cytokines in circulation as well as in BAT and WAT. Moreover, these AT cytokines appear to be derived exclusively from macrophages residing in these tissues. Adipose tissue atrophy in cancer patients with CACS has been associated with an increase in subcutaneous AT macrophages [65–67] and tissue inflammation [66–68]. Although, macrophage infiltration has also been described in WAT from tumor-bearing rodents [10, 66, 69], to our knowledge this is the first report of macrophages as the source of pro-inflammatory cytokines in adipose tissue in CACS. These findings may explain why AT remains an important source of pro-inflammatory cytokines even when the adipocyte mass is significantly reduced in CACS. Also, this may be clinically relevant to cancer patients since knowing the source of inflammation may allow us to target these pathways more effectively [70]. Future studies should consider IHC staining of crown-like structures (dying adipocytes surrounded by macrophages) to further characterize this process in AT [65].

Ghrelin is thought to have anti-inflammatory effects in other settings [71–73] but this is not yet clear in CACS. Some reports suggest an anti-inflammatory effect of native ghrelin administration, but this was not confirmed in other studies using GHSR-1a agonists [16, 43]. Here we report that ghrelin modulates inflammation in a tissue-specific manner. Ghrelin did not prevent tumor-induced increases in circulating inflammatory cytokines or in BAT IL-1β or MCP-1 protein levels. However, it mitigated LLC-induced inflammation in WAT. This effect was seen in both genotypes although it was clearer in wild type animals partly because *Ghsr*^−/−^ mice appear to be resistant to tumor-induced inflammation. GHSR-1a is not expressed in adipocytes [22] but is present in macrophages [74] and our findings are consistent with a previous report showing that old, non-tumor-bearing *Ghsr*^−/−^ mice have reduced macrophage infiltration, a shift on macrophage differentiation towards a more anti-inflammatory phenotype, and decreased inflammation in adipose tissue [75]. However, a GHSR-1a-independent effect of ghrelin on macrophages is also possible as it has been proposed in other studies [76–78]. Taken together, our data is consistent with a WAT-specific, anti-inflammatory effect of ghrelin that is partly GHSR-1a dependent. This is clinically relevant as GHSR-1a agonists are in clinical development for CACS and their effect on these GHSR-1a independent pathways is not known [15]. Also, the differences we report between serum, WAT and BAT levels underscore the limitations of relying exclusively on circulating cytokine levels when trying to determine the potential role of inflammation in other tissues.

In BAT, macrophage infiltration contributes to the high levels of inflammatory cytokines (TNF, IL-6, and IL-1β) in conditions associated with AT hypertrophy such as high fat diet [79, 80] or obesity [81, 82]. In CACS, the aforementioned tumor-induced inflammation is thought to play an important role in BAT thermogenesis [10, 83]; however, the source of inflammation in BAT is not known. Similar to WAT, we found that BAT IL-6 and TNF come exclusively from macrophages in the setting of cachexia. However, their expression in BAT were lower than in WAT and no significant changes were found in response to tumor implantation or ghrelin. We found a significant tumor-effect on increasing IL-1β levels in BAT although ghrelin did not prevent this increase, suggesting tissue-specific differences in inflammation between BAT and WAT in response to tumor and ghrelin. Our data also suggests that WAT is a significant source of inflammatory cytokines, which express the highest levels of IL-1β, IL-6, and TNF when compared to BAT and circulating levels.

Energy expenditure is an important mechanism in the regulation of body weight and is increased in CACS [36, 43, 84]. Factors contributing to EE include physical activity and resting EE (REE) [85, 86] and adipose tissue can lead to an increase in REE by uncoupling oxidative phosphorylation in mitochondria thereby releasing heat through activation of a proton leak [87, 88]. In WAT, browning has been noted in multiple cancer cachexia animal models and in small human studies with adipocytes showing an upregulation of the main regulator of thermogenesis, UCP1 [38, 89]. In BAT, increased thermogenesis has been reported in cachectic animals independently of decreased food intake [30] or their ability to maintain their body temperature [83] and in humans [10, 90]. Proinflammatory cytokines have been suggested as key drivers of WAT browning [10, 38] and of BAT thermogenesis through activation of sympathetic nervous system or targeting BAT directly [83, 91–93]. Here we show that LLC-tumor implantation led to an increase in total EE in spite of a significant decrease in physical activity, suggesting an increase in REE. In WT animals, this increase in EE can be accounted for by the tumor mass whereas the tumor only partially explains the increases in EE seen in KO mice, suggesting that other mechanisms are at play. Although this study was not set to established the mechanisms behind this increase in EE seen in KO mice after tumor implantation, we hypothesize that the increase in white and brown adipose tissue thermogenesis may be at least partly responsible for the differences between genotypes. The fact that exogenous ghrelin administration did not prevent these changes suggest that other ligands may be responsible for these GHSR-mediated effects. More studies would be required to test this hypothesis. The effect of ghrelin or GHSR1a agonists on energy expenditure is unclear with some studies showing a decrease in EE [94, 95] while others showed no effect [96–98]. In this study, we did not see a significant effect of ghrelin on preventing LLC-induced fat browning, BAT thermogenesis, increased REE or decreased physical activity in the setting of CACS despite the fact that ghrelin prevented fat and weight loss and anorexia. We hypothesize that differences in the models, route of administration and treatment regimen and agents used (LLC mice *vs*. C26 mice or hepatoma model in rats, administration *via* s. q. *vs*. oral gavage *vs*. osmotic mini pump, ghrelin *vs*. GHSR-1a agonists) could account for these discrepancies. Future studies will be needed to test this hypothesis.

There were limitations to our approach. This study was not set up to establish the safety of ghrelin administration in the setting of cancer. Nevertheless, none of the studies published to date using ghrelin or GHSR-1a agonists in mice or humans have shown an increase in tumor progression [99]. The extent to which brown adipose tissue and browning of WAT contribute to the phenotype of cachexia and increased energy expenditure is not well-characterized in humans as there are only a few studies showing browning of adipose tissue in cancer cachexia patients [38, 89]. Clinical studies will be needed to establish the clinical significance of our findings in this rodent model. These experiments were not designed to characterize other mechanisms contributing to the protective role of GHSR-1a in this study. Moreover, our data suggest that there is an alternative receptor for ghrelin although identification of this receptor remains elusive and is the focus of other studies. Lastly, we used a global GHSR-1a KO line in the current study, which may limit our understanding of tissue- and developmental-specific differences, and the LLC tumor model, although well-characterized and native to our mouse strain, has a high tumor burden compared to human disease.

In summary, ghrelin prevents LLC tumor-induced body weight and fat loss by a combination of GHSR-1a-dependent mechanisms including preventing anorexia, and other mechanisms that are partly GHSR-1a-independent such as WAT lipolysis. The increase in inflammation in AT induced by tumor implantation is prevented by ghrelin only in WAT; however, tumor-induced WAT browning, and increased BAT inflammation, uncoupling and whole body energy expenditure are not prevented by ghrelin even when the presence of GHSR-1a appears to contribute to maintaining energy balance in the present study. Tumor-induced WAT browning and BAT thermogenesis are associated with significant increases in REE and these seem to be independent of inflammation given that downregulating it does not prevent these changes. These results are clinically relevant because they show that ghrelin ameliorates WAT inflammation, lipolysis and fat atrophy in CACS in spite of not having a discernible effect on energy expenditure, WAT browning or BAT inflammation and thermogenesis. Our data fills an important gap in the knowledge regarding the mechanisms of action of ghrelin in cancer cachexia and should inform the design of future preclinical and clinical studies targeting this pathway.

## MATERIALS AND METHODS

### Animals

Five to seven-month-old male C57BL/6J growth hormone (GH) secretagogue receptor wild type (*Ghsr*^+/+^) and knockout (*Ghsr*^−/−^) congenic mice were used for all experiments. Briefly the *Ghsr*^+/+^ and *Ghsr*^−/−^ mice were originally from Dr. Roy G. Smith PhD’s laboratory [23] and the *Ghsr*^−/−^ mice were backcrossed with C57BL/6J for at least 10 generations to minimize selective genetic traits. The mice used in the study were offspring of these congenic mice and were bred in the Animal Research Facilities at the Veterans Affairs Puget Sound Health Care System. Mice were individually housed, acclimated to their cages and human handling for 1 week before the experiments and maintained on a 12/12 light/dark cycle (lights on at 6AM). All experiments were conducted with the approval of the Institutional Animal Care and Use Committee at VA Puget Sound Health Care System and were in compliance with the NIH Guidelines for Use and Care of Laboratory Animals. Sample sizes of each experiment are shown in the figure legends.

### Tumor implantation and ghrelin administration

The procedures of tumor implantation (TI) and ghrelin intervention were described previously [16]. In brief, mice were injected subcutaneously (s. q.) with Lewis lung carcinoma (LLC) cells (1 × 10^6^ cells, CRL1642, American Type Culture Collection, Manassas, VA, USA) into the right flank or with equal volume and number of heat-killed LLC cells (HK). Approximately 7 days after tumor implantation (TI), when the tumor was palpable (~1 cm in diameter), the tumor-bearing mice were treated with either acylated ghrelin (AS-24160, Anaspect, Fremont, CA, USA) at a dose of 0.8 mg/kg or vehicle (0.9% sodium chloride, 8881570121, COVIDIEN, Dublin, Ireland), s. q., twice daily, while mice in HK+V group received vehicle (saline, same volume), s. q., twice daily for two weeks. Our group and others have looked at the effect of different treatment regimens of ghrelin ranging from 0.08 to 8 mg/kg and from one day to 45 days [16, 100]. The dose selected for this study has been shown to be well-tolerated and to induce a 5–10% weight gain, a 20–30% increase in fat mass and a ~10% increase in food intake [100]. These changes are similar to those seen in recent human trials where ghrelin mimetics are being used highlighting its translational and clinical relevance [15, 42]. Food and water were given ad libitum except for pair-fed groups (T+G+P) used when measures of lipolysis in WAT were obtained. Pair-fed mice were treated the same as T+G mice and were pair-fed to their T+V peers with the same genotype. Mice were euthanized by CO_2_ on Day 21 after TI, approximately 2 weeks after TN. Blood samples were collected and then processed into plasma. Fat pads including iWAT, eWAT, and BAT, as well as tumors were collected and their wet weight measured during dissection. The timeline of the study is demonstrated in Supplementary Figure 4.

### Body weight, food intake, and body composition

Body weight and food intake were assessed daily starting before TI (baseline) until endpoint. Parameters of body composition, including LBM and fat mass (FM) were measured by nuclear magnetic resonance (NMR, Bruker optics, The Woodlands, TX, USA) and identified at baseline before tumor implantation, when tumor was noted, and 2 weeks after tumor noted before terminating the experiment (endpoint). The body weight changes are expressed as carcass weight at the endpoint (body weight at endpoint minus tumor mass) normalized to baseline body weight in %. Fat mass measured by NMR and fat pad weights collected at endpoint were normalized to baseline fat mass measured by NMR at baseline.

### Comprehensive laboratory animal monitoring system (CLAMS™)

The Comprehensive Laboratory Animal Monitoring System (CLAMS™, Columbus Instruments, Columbus, OH, USA) was used to identify metabolic parameters of the animals as we previously described [101]. *Ghsr*^+/+^ and *Ghsr*^−/−^ mice were individually housed in CLAMS cages for 84 hours before TI (baseline) as well as at the endpoint (see the Supplementary Figure 4, timeline for the study). The first 12 hours of CLAMS was considered as the acclimation phase and the data for the next 72 hours were analyzed. Food intake (g), oxygen consumption (VO_2_) (mL/h), carbon dioxide production (VCO_2_) (mL/h), and locomotor activity (infrared beam-break counts) were recorded automatically by the CLAMS system every 20 min. The respiratory exchange ratio (RQ) and energy expenditure (EE, or heat generation) were calculated from VO_2_ and VCO_2_ gas exchange data as follows: RQ = VCO_2_/VO_2_ and EE = (3.815 + 1.232 × RQ) × VO_2_, respectively. EE was analyzed by normalizing to the endpoint LBM with or without tumor. Locomotor activity was measured on x- and z-axes by the counts of beam-breaks during the recording period. The data shown in the results was summarized as the mean of every 6 hours in a 72-hour-period.

### Western blot analyses

iWAT was homogenized in 20 mM 3-(N-morpholino) propanesulfonic acid (MOPS), 2 mM ethylene glycol-bis (β-aminoethyl ether)-N, N, N′, N′-tetraacetic acid (EGTA), 5 mM Ethylenediaminetetraacetic acid (EDTA), 30 mM sodium fluoride, 40 mM β-glycerophosphate, 10 mM sodium pyrophosphate, 2 mM sodium orthovanadate, 0.5% NP-40 and complete protease inhibitor cocktail (Roche, Nutley, NJ, USA) and centrifuged at 13,000 g for 20 min at –3°C. The supernatant was collected while carefully avoiding the lipid layer on top. Protein concentration was then measured with a bicinchoninic acid (BCA) protein quantification kit (Thermo Scientific, Waltham, MA, USA). Protein extract was loaded on 4–12% NuPAGE gels (Invitrogen, Carlsbad, CA, USA) and blotted onto Immobilon FL polyvinylidene fluoride or polyvinylidene difluoride (PVDF) membranes (Millipore, Billerica, MA, USA). Membranes were blocked at room temperature for 1 h in Odyssey LI-COR Blocking Buffer (LI-COR, Lincoln, NE, USA) 1:1 diluted in tris-buffered saline (TBS) and incubated in primary antibodies in 1:1 Blocking Buffer/TBS-T overnight at 4°C. Primary antibodies against adipose triglyceride lipase (ATGL), phospho–hormone sensitive lipase (HSL) (Ser563), glyceraldehyde 3-phosphate dehydrogenase (GAPDH, Cell Signaling Technology, Beverly, MA, USA). Due to the limited amount of available iWAT tissue, we selected GAPDH as our loading control for both HSL (Ser 563) and ATGL. After 3× 5 min washes in TBS-T (0.1%), the blots were incubated with Dylight 680-conjugated goat anti-rabbit IgG and Dylight 800-conjugated goat anti-mouse IgG (both Thermo Scientific, Waltham, MA, USA) for 1 h at room temperature in blocking buffer containing 0.1% TBS-T and 0.1% SDS. After three washes in TBS-T and a final wash in TBS, we scanned the blots with the LI-COR Odyssey (LI-COR, Lincoln, NE, USA) and quantified them with Odyssey 3.0 software on the basis of direct fluorescence measurement.

### Electrochemiluminescence immunoassay

Inflammatory cytokines IL-1β, IL-6, and TNF-α and macrophage marker MCP-1 in iWAT, BAT, and serum were detected by U-PLEX Biomarker Group1 (ms) Assays which are developed by Meso Scale Diagnostics (K15069L-1, MSD, Rockville, MD, USA). A protocol provided by manufacturer was used for this assay. In brief, each plate was prepared by overnight coating with the multiplex coating solution at 4 °C, which contained linker-coupled biotinylated antibodies. Standards and serum samples were diluted with Diluent 41 into 2-fold and loaded onto the coated plate on the next day. For iWAT and BAT samples, 150 ug of the protein lysate was diluted with Diluent 41 and loaded onto each well. The plate was incubated at room temperature (RT) with shaking for 2 h followed by 3 times of wash in phosphate buffered saline with 0.05% Tween 20 (PBS/T). Sulfo-tag labeled detection antibody was then added to plates and incubated for 2.5 h. After another 3 washes in PBS/T, Read Buffer T (2×) was added and the plate was read on MSD Sector Imager (MSD).

### Immunohistochemistry

The iWAT and BAT were mounted with Optimal Cutting Temperature (OCT) compound (VWR 25608-930, VWR, Radnor, PA, USA) and flash frozen in liquid nitrogen-chilled isopentane immediately after tissue collection. The OCT-mounted iWAT and BAT blocks were sliced at 14 μm using a Cryostat (Leica CM3050S, Nussloch, Germany) at –40°C. Before the process of staining, slides were dehydrated at RT for 30 minutes followed by incubating in methanol for 15 minutes at –20°C. Hematoxylin and eosin (H&E) staining was performed on iWAT and BAT slides to detect morphological changes. To identify the colocalization of F4/80 and IL-6 or TNFα in iWAT and BAT, slides were blocked with 10% donkey serum for 1 hour at RT and followed by incubating in primary antibodies (F4/80 Monoclonal Antibody 1:100, MF48000, Thermo Fisher Scientific; Anti-IL-6 antibody 1:100, ab6672, Abcam; TNF alpha monoclonal antibody, FITC, eBioscience™ 1:200, 11-7349-82, Thermo Fisher Scientific) at 4°C for overnight. After 3 washes in PBS, the slides were incubated by the corresponding secondary antibodies (Alexa Fluor 594 donkey anti-rat IgG, A21209, or Alexa Fluor 488 donkey anti-rat IgG, A21208, for F4/80; Texas Red goat anti-rabbit IgG, T-2767, for IL-6) for 2 hours at RT and followed by incubating in 1:1000 DAPI (62248, Thermo Fisher Scientific) in PBS for 1min. The slides were then mounted by Prolong Gold AntiFade reagent (P36930, Thermo Fisher Scientific) with coverslips. To identify UCP1 in iWAT and BAT, slides were incubated with 3% hydrogen peroxide (323381, Sigma-Aldrich, St. Louis, MO, USA) for 30 min and then in 2.5% normal horse serum for 1 hr. Then the slides were incubated with UCP1 Polyclonal Antibody (PA1-24894, Thermo Fisher Scientific) diluted 1:200 in 2.5% normal horse serum at 4°C for overnight. On the following day, signals were visualized using SignalStain^®^ Boost IHC Detection Reagent (8114, Cell Signaling) and the SignalStain^®^ DAB Substrate kit (8059, Cell Signaling). The stained slides were dehydrated by 70%, 90%, 100% ethanol, and 100% xylene sequentially and mounted with coverslips by using Permount (SP15-100, Thermo Fisher Scientific). All stained slides were imaged by Nikon NiE microscope at 20× (iWAT) or 40× (BAT). The positive cells (immunofluorescence) or positive area (DAB stain) in the section were quantified and normalized to the total area of the section (mm^2^) using ImageJ analysis software (National Institutes of Health, https://imagej.nih.gov).

### Statistics

Two-way ANOVA was performed to identify differences between genotypes (*Ghsr*^+/+^ *vs*. *Ghsr*^−/−^) across treatments (HK+V, T+V, T+G, and T+G+P [for lipolysis analysis]) followed by Fisher’s LSD post hoc tests. For inflammatory cytokines, Kruskal–Wallis test was performed to identify the differences between groups. Values are presented in mean ± SEM. All statistical testing was performed using IBM SPSS version 18 software. Significant difference was set at ^*^ *p* < 0.05; ^**^ *p* < 0.01; ^***^ *p* < 0.001.

## SUPPLEMENTARY MATERIALS



## Abbreviations

AT: adipose tissue
GHSR-1a: growth hormone secretagogue receptor-1a
LLC: Lewis Lung Carcinoma
iWAT: inguinal white adipose tissue
eWAT: epididymal white adipose tissue
BAT: brown adipose tissue
KO: knockout
WT: wild type
HK: heat-killed
NMR: nuclear magnetic resonance
FI: food intake
ATGL: adipose triglyceride lipase
HSL: hormone sensitive lipase
IL-1β: Interleukin-1β
IL-6: Interleukin-6
TNF: tumor necrosis factor
MCP-1: monocyte chemoattractant protein-1
UCP-1: uncoupling protein-1
ATP: adenosine triphosphate
IHC: immunohistochemistry
EE: energy expenditure
LBM: lean body mass
CLAMS: comprehensive laboratory animal monitoring system
RQ: respiratory exchange ratio
CACS: cancer anorexia and cachexia syndrome
FDA: Food and Drug Administration
TAG: triacylglycerol
PKA: protein kinase A
MAPK: mitogen-activated protein kinase
REE: resting energy expenditure
GH: growth hormone
TI: tumor implantation
MOPS: 3-(N-morpholino) propanesulfonic acid
EGTA: ethylene glycol-bis(β-aminoethyl ether)-N,N,N′,N′-tetraacetic acid
EDTA: ethylenediaminetetraacetic acid
BCA: bicinchoninic acid
PVDF: polyvinylidene fluoride or polyvinylidene difluoride
TBS: tris-buffered saline
GAPDH: glyceraldehyde 3-phosphate dehydrogenase
PBS: phosphate-buffered saline
OCT: Optimal Cutting Temperature
FITC: fluorescein isothiocyanate
DAPI: 4’,6-diamidino-2-phenylindole
DAB: 3,3′-Diaminobenzidine
