# Supplementary material for: Ghrelin ameliorates tumor-induced adipose tissue atrophy and inflammation via Ghrelin receptor-dependent and -independent pathways
Source: Oncotarget. 2020 Sep 1;11(35):3286–302. doi: 10.18632/oncotarget.27705 (PMC7476735; doi:10.18632/oncotarget.27705)
Supplement: Supplementary file 1 [file oncotarget-11-3286-s001.pdf]

Ghrelin ameliorates tumor-induced adipose tissue atrophy and inflammation *via* Ghrelin receptor-dependent and -independent pathways

SUPPLEMENTARY MATERIALS

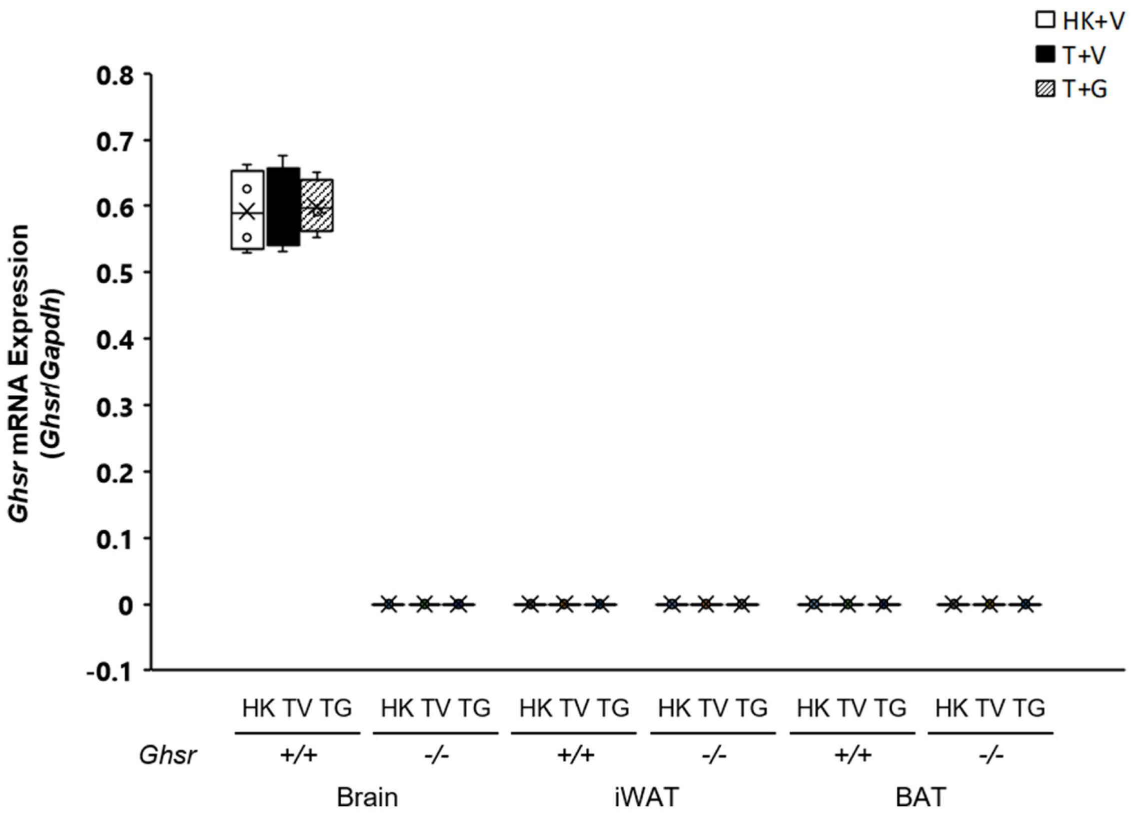

Supplementary Figure 1: Gene expression of *Ghsr* in brain, iWAT, and BAT in *Ghsr*$^{+/+}$ and $^{-/-}$ mice. Data is expressed as box-and-whisker plot showing the median (middle line), mean (middle cross), upper and lower quartiles (box), maximum and minimum (whiskers). Relative gene expression was determined by normalization to *Gapdh*. $N = 4$/group. *Ghsr* was only detected in brain in *Ghsr* $^{+/+}$ mice. No *Ghsr* expression was detected in any tissue in *Ghsr*$^{-/-}$ or adipose tissue in *Ghsr*$^{+/+}$ mice.

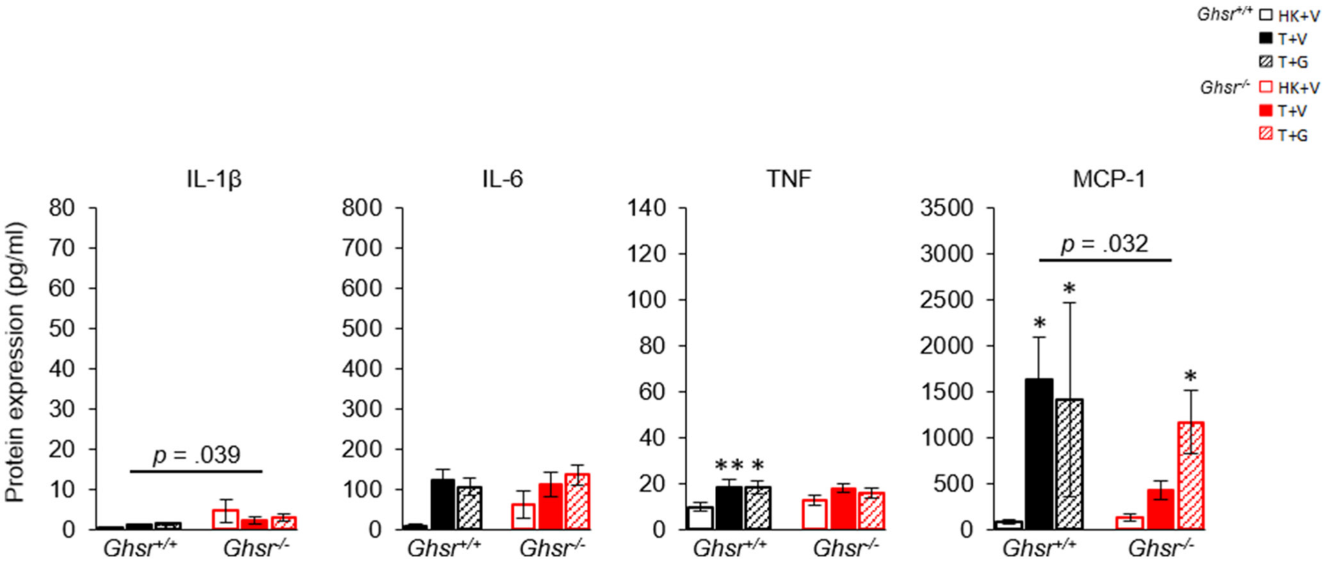

Supplementary Figure 2: Effects of ghrelin on LLC-induced protein-level changes in inflammation (IL-1β, IL-6, and TNF) and macrophages (MCP-1) in plasma (pg/ml, *n* = 11–14). Two-way ANOVA was performed to detect genotype and treatment differences. *,**: different than HK+V within the same genotype (*$p < .05$; **$p < .01$). Genotype effects are shown in p-values above the corresponding figures ($p < .05$). Data are shown as mean ± SE.

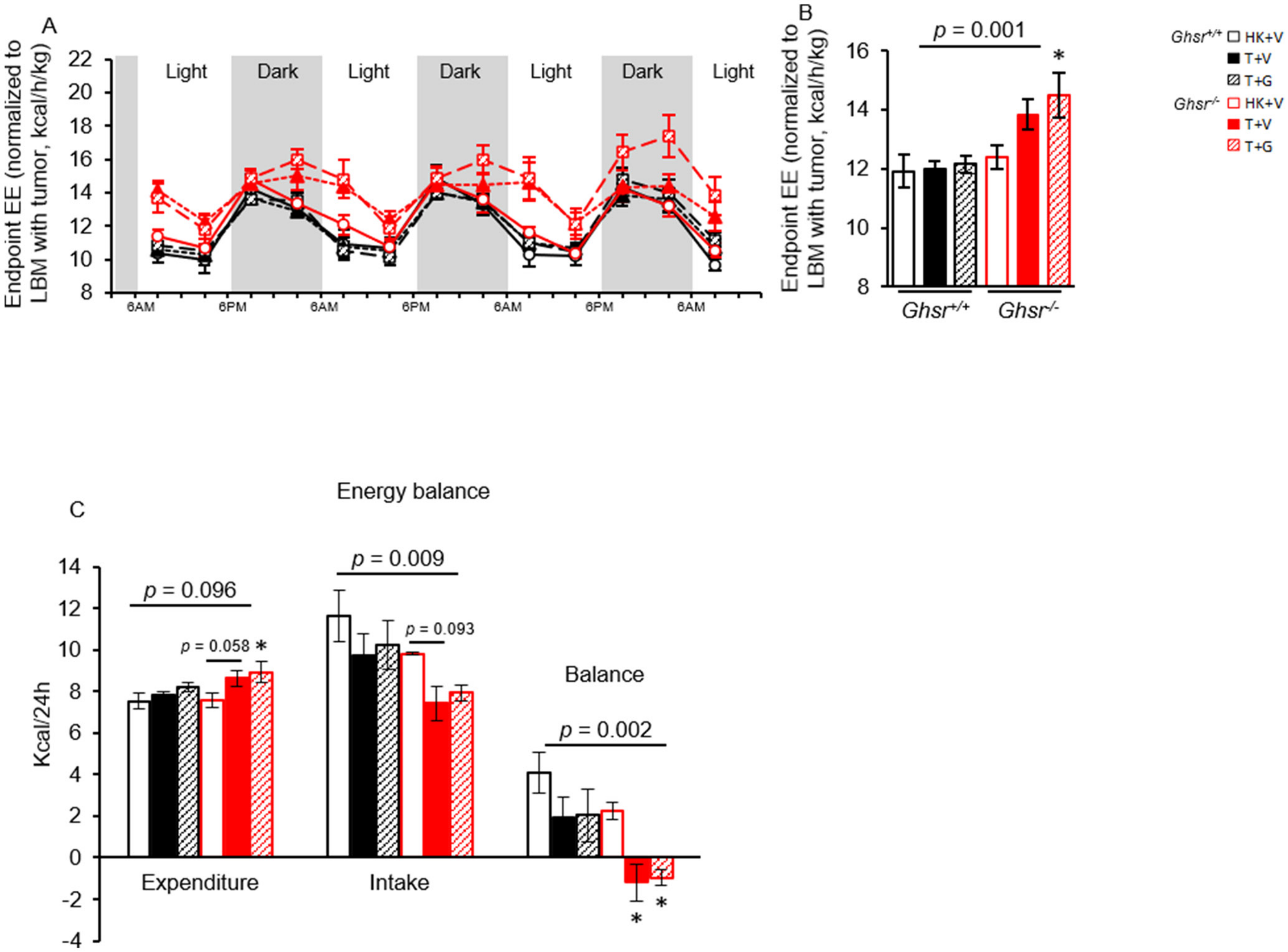

Supplementary Figure 3: Energy expenditure (including the tumor) and balance. Indirect calorimetry measurements by CLAMS. HK+V: heat-killed + vehicle; T+V: tumor + vehicle; T+G: tumor + ghrelin. (**A**–**B**) Energy expenditure (EE) adjusted by LBM (with tumor) is expressed as kcal/h/kg (A) during 72 hours before sacrificing (after 2 weeks of ghrelin treatment); (B) Average daily EE over the 72 hours before sacrificing. (**C**) Energy balance in each experimental group. Energy expenditure at endpoint [daily average (kcal/24 h) during 72 hours before sacrificing], energy intake [daily average (kcal/24 h) during 72 hours before sacrificing, calculated by food intake (g) × 3.07 kcal/g], and energy balance (kcal difference between energy intake and expenditure). Two-way ANOVA was performed to detect genotype and treatment differences. $^{*}p < 0.05$ compared to HK+V within the same genotype. Genotype effects are shown in *p*-values above the corresponding figures ($p < 0.05$). $N = 4$ for HK+V groups and $N = 6$ for the rest of the groups. Data are shown as mean ± SE.

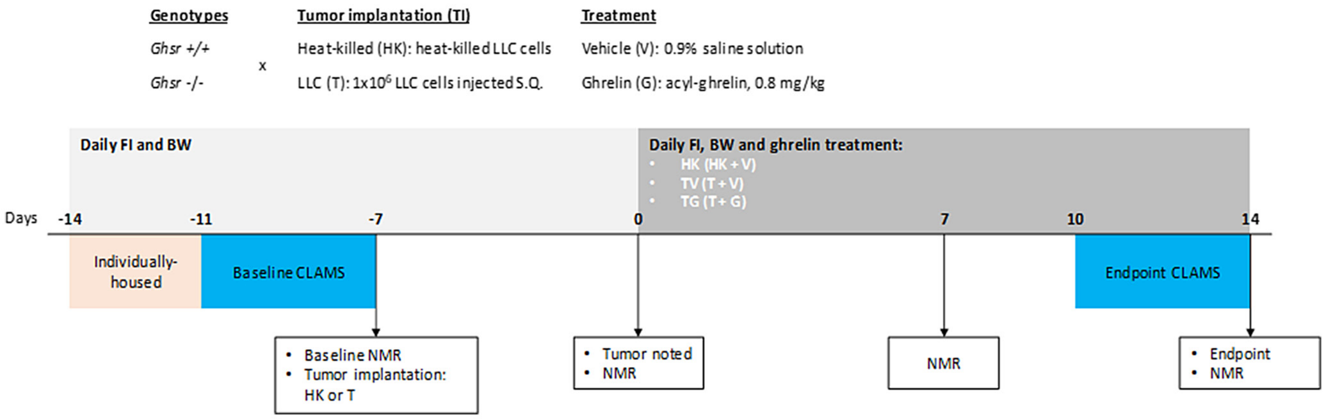

Supplementary Figure 4: Timeline of current study. *Ghsr*$^{+/+}$ and $^{-/-}$ mice were injected with LLC (T, 1×10^6 cells, s.q.) into the right flank or with equal volume and number of heat-killed LLC cells (HK+V). Approximately 7 days after tumor implantation, when the tumor was palpable (day 0), the tumor-bearing mice were treated with either acylated ghrelin, 0.8 mg/kg (T+G) or vehicle (0.9% sodium chloride, T+V), s.q., twice daily, while mice in HK+V group received vehicle (saline, same volume), s.q., twice daily for two weeks. Body composition were identified by NMR before tumor implantation (7 days before tumor noted, baseline) and weekly till the endpoint. All the mice were individually housed in CLAMS cages for 84 hours before TI (11–7 days before tumor noted, baseline) as well as at the endpoint (day 10–14 after tumor noted).
